# Development and validation of a disease-specific health-related quality of life questionnaire for alcohol-associated liver disease: The CLDQ-ALD

**DOI:** 10.1097/HC9.0000000000000963

**Published:** 2026-05-26

**Authors:** Zobair M. Younossi, Maria Stepanova, Issah Younossi, Leyla deAvila

**Affiliations:** Center for Outcomes Research in Liver Disease, Washington, District of Columbia, USA

**Keywords:** alcohol use disorder, chronic liver disease, disease burden, emotional well-being, fatigue, patient-reported outcomes, pruritus

## Abstract

**Background:**

Accurate assessment of health-related quality of life (HRQL) is essential in clinical research and therapeutic trials in alcohol-associated liver disease (ALD). The study aimed to develop and validate an ALD-specific HRQL instrument using a standard protocol for disease-specific HRQL instrument development.

**Methods:**

A comprehensive list of HRQL items was generated based on ALD patients’ individual interviews and focus groups, existing HRQL instruments, and input from expert health care providers. Items were reduced through administration to patients with ALD, calculation of item impact scores, assessment of face validity, exploratory factor analysis, and evaluation of within-domain consistency and redundancy. The final Chronic Liver Disease Questionnaire (CLDQ)-ALD instrument underwent initial steps for psychometric validation.

**Results:**

The initial questionnaire comprised 90 items. Across 2 rounds of item reduction, it was completed by 72 patients with ALD (32% <45 y, 69% male, 18% with T2D, 39% cirrhosis, 40% current alcohol use) and reduced to 40 final items. Exploratory factor analysis identified 9 factors explaining 95% of the total variance. Based on the factor loadings, items were assigned to 9 domains (Fatigue, Alcohol, Function, Physical, Abdominal Symptoms, Itching, Sleep, Emotional, and Worry). Face and construct validity were assessed, along with concordance with another validated CLDQ instrument (CLDQ-metabolic dysfunction–associated steatohepatitis). The final CLDQ-ALD demonstrated good to excellent psychometric performance, with high internal consistency (Cronbach α >0.85 across all domains and >0.90 in 5 of 9 domains). Known-groups validity demonstrated mean score differences >0.5 (on a scale 1–7) in relevant domains by sex, presence of T2D, current alcohol use, depression, clinically overt fatigue, and cirrhosis.

**Conclusions:**

CLDQ-ALD version 1 is a disease-specific HRQL instrument for use in clinical research and therapeutic trials involving patients with ALD.

## Graphical abstract

**Figure:**
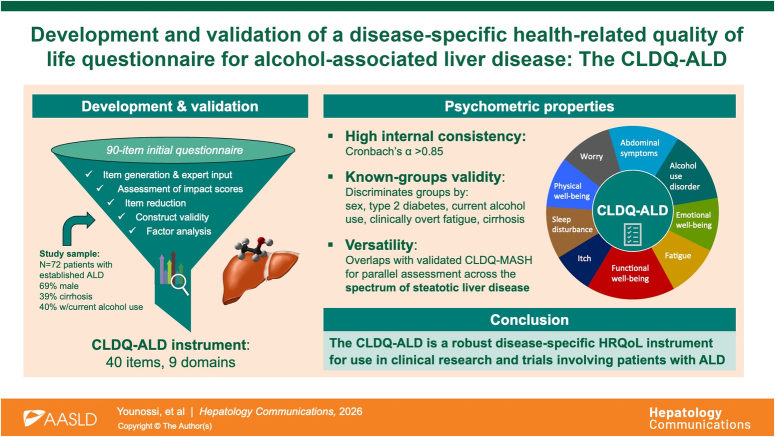

## INTRODUCTION

Alcohol-associated liver disease (ALD) remains a major global cause of chronic liver disease, cirrhosis, and liver-related death, and in recent years has become a leading indication for liver transplantation in both the United States and Europe.^1^,^2^,^3^,^4^ Recent epidemiologic data also suggest that ALD burden is rising in several regions, including among younger adults, with important implications for long-term morbidity, transplant needs, and health care utilization.^1^,^2^,^3^

Beyond mortality, ALD contributes substantially to disability and health loss at a population level, as reflected by global estimates of alcohol-attributable cirrhosis and liver cancer deaths and disability-adjusted life years.^5^,^6^,^7^ At the patient level, ALD is associated with a high symptom burden and functional impairment. Indeed, patients commonly experience fatigue, sleep disturbance, pain, pruritus, emotional distress, cognitive complaints (including those related to HE), and reduced ability to perform usual activities, which collectively impair their health-related quality of life (HRQL) and other patient-reported outcomes.^8^,^9^,^10^,^11^ Importantly, these impairments are not limited to end-stage disease, with HRQL deficits having been documented across the ALD spectrum, including alcohol-associated hepatitis and compensated or decompensated cirrhosis.^8^,^12^

The ALD also carries a distinctive psychosocial context. Stigma related to alcohol use and perceived “self-inflicted” disease can affect patients’ mental well-being, health care experiences, and engagement with treatment, creating an additional dimension of disease burden that may not be fully captured by generic HRQL measures.^13^ Furthermore, alcohol use disorder and comorbid depression/anxiety are prevalent in patients with ALD and can independently worsen patient-perceived health status, complicate symptom interpretation, and influence outcomes.^9^,^10^,^11^,^14^

Although HRQL in ALD has been assessed using generic instruments (eg, SF-36) and liver disease-specific instruments (eg, Chronic Liver Disease Questionnaire [CLDQ], LDQoL, PROMIS-based metrics), generic tools may lack sensitivity to detect clinically meaningful changes in ALD-specific symptom patterns and psychosocial impacts.^8^,^10^,^11^,^12^ The original Chronic Liver Disease Questionnaire (CLDQ) was developed as a liver-specific HRQL instrument for chronic liver disease broadly, but it was not designed to comprehensively address etiology-specific issues unique to ALD.^15^ Over time, this limitation has motivated the development of disease-specific “CLDQ family” instruments for distinct liver diseases (eg, NAFLD/NASH, metabolic dysfunction–associated steatohepatitis [MASH], HCV, HBV, PSC, PBC), reflecting increasing expectations that patient-reported outcome instruments used in clinical research demonstrate clear content validity in the target population and capture outcomes that matter most to patients.^16^,^17^,^18^,^19^,^20^,^21^,^22^ Regulatory guidance similarly emphasizes that patient-reported outcome endpoints intended to support labeling claims should be developed and validated with explicit attention to the target population and conceptual framework.^23^

Accordingly, the aim of this study was to develop and validate an ALD-specific version of the CLDQ CLDQ-ALD version 1 (CLDQ-ALD) for use in epidemiologic studies, clinical trials, and routine clinical practice, enabling accurate assessment of the symptom burden and HRQL impacts that are most relevant to patients living with ALD.

## METHODS

### Study sample

For this study, subjects with an established diagnosis of ALD were recruited from the Global Liver Registry sites. For eligibility, ALD was defined by a self-reported established diagnosis of alcohol-related (or alcohol-associated) liver disease. All included subjects were required to be at least 18 years of age and to be willing and able to provide informed consent for the study. Subjects were not eligible for inclusion in the study if they had any condition that, in the opinion of the principal investigator, would make the subject unsuitable for enrollment or that could interfere with the subject’s participation (such as major psychiatric or emotional problems, language, or cognitive difficulties); had any other cause of chronic liver disease (eg, viral hepatitis, autoimmune liver disease); had a history of liver transplantation; or were unwilling or unable to provide an informed consent.

Subjects’ basic demographic and clinical parameters (age, sex, weight, height, current alcohol use [any amount], history of type 2 diabetes, cirrhosis, select comorbidities) were collected upon enrollment. All included subjects completed either a long list of questions (item selection questionnaire) or a questionnaire with a reduced list of candidate questions (CLDQ-ALD version 0).

All research was conducted in accordance with both the Declarations of Helsinki and Istanbul, the research was approved by our institutional review board (WCG IRB) and written consent was given by all subjects.

### Step 1: Development of CLDQ-ALD

#### Item selection and reduction

A long-item selection questionnaire was developed to include HRQL items from other CLD-specific instruments, as well as feedback from ALD patient interviews and ALD patients’ focus groups, and input from health care providers who routinely treat patients with ALD. That initial 90-item questionnaire was administered to the first 20 subjects with ALD. To determine the Impact Score of each item, patients with ALD were asked to grade how much a particular problem bothered them, on a scale from 0 (“not at all”) to 5 (“very much”). To reduce the number of items from the long-item selection questionnaire, the proportion of patients who indicated that a problem bothered them “not at all” or “a little” (low impact) was calculated for each item. Similarly, the proportion of patients who reported that a problem bothered them at least “quite a bit” (high impact) was also calculated. The items with the proportion of low-impact answers <70% and the items with the proportion of high-impact answers ≥15% were considered impactful. These impactful items were supplemented with pertinent items retained from other CLDQ instruments (to facilitate harmonized HRQL assessment across the spectrum of steatotic liver disease) and with additional items retained to ensure adequate representation and internal consistency of clinically relevant disease-specific domains (eg, HRQL impact due to itching, alcohol use/recovery). This item selection process resulted in the CLDQ-ALD version 0.

#### Factor analysis

The CLDQ-ALD version 0 was administered to a larger group of ALD subjects with the aim of developing a final CLDQ-ALD instrument that would include no more than 40 items grouped into easily interpretable domains.

Exploratory factor analysis was conducted using the items of CLDQ-ALD version 0. The only restriction to the factor analysis output was that each domain must include 2 or more items, each with a loading of at least 0.50, to reduce the effect of sporadic, haphazard responses to individual questions. The number of factors was not predefined but rather determined from the proportion of variance accounted for by the factors (95% or more but not exceeding 100%). Factor loadings were used to assign items to domains, followed by assessment of face and construct validity, elimination of redundancy (to meet the target instrument size), and concordance with the validated CLDQ-MASH. This domain design process resulted in the final CLDQ-ALD version 1.

#### Final CLDQ-ALD instrument

The items of CLDQ-ALD were distributed into domains (according to the factor loadings and the content validity assessment) so that each item would be assigned to one domain. In the final CLDQ-ALD instrument, each domain includes at least 2 items, and the final domain names were chosen to reflect their primary concept.

Similar to other instruments of the CLDQ family, the items of CLDQ-ALD were formulated as follows: “How much of the time/How often during the past 2 weeks you <experienced a problem>/<were bothered by a problem> ?”. A 1–7 Likert scale was used for the responses, with the score of 1 corresponding to “all of the time,” and the score of 7 to “none of the time.” Again, similar to other CLDQ instruments, the scoring scheme for CLDQ-ALD requires each domain score to be calculated as an average of the domain’s items, without item weighting or additional Likert scores transformations. Finally, similar to other CLDQ instruments, greater scores for all CLDQ-ALD domains would reflect better health and HRQL.

### Step 2: Validation of CLDQ-ALD

For the second step, a standard HRQL instrument validation pipeline was applied to validate the CLDQ-ALD instrument.

#### Internal consistency

Internal consistency of CLDQ-ALD was assessed by the calculation of Cronbach’s alpha coefficients for the domains, and by the calculation of item-to-own-domain correlations for all items. The distributions of the domain scores were also qualitatively evaluated for skewness and floor/ceiling effects. Discriminant validity assessment was run with the purpose of confirming that each item was the most correlated with its own domain.

#### Known-groups validity

Known-groups validity was assessed by evaluation of the association of CLDQ-ALD domain scores with potentially relevant demographic and clinical parameters. The validation parameters were age, gender, current alcohol use, history of type 2 diabetes, depression, clinically overt fatigue, and cirrhosis (all parameters self-reported). Group differences in CLDQ-ALD domain scores were evaluated using the Wilcoxon nonparametric test. In addition, differences in mean domain scores were calculated to complement hypothesis testing by characterizing the magnitude and direction of the group differences.

All analyses were run using SAS 9.4 (SAS Institute). All research was conducted in accordance with both the Declarations of Helsinki and Istanbul, was approved by the IRB or other supervisory institutions at each participating site, and informed consent was obtained from all subjects.

## RESULTS

There were 72 subjects included in the study: 32% below 45 years, 69% male, 18% with type 2 diabetes, 39% with cirrhosis, and 40% reported current alcohol use (any amount) (Table 1).

**TABLE 1:** Demographic and clinical parameters of subjects with ALD used for the development of CLDQ-ALD

	N=72
Age <45 y, n (%)	23 (32)
Age 45–54 y, n (%)	23 (32)
Age >55–69 y, n (%)	26 (36)
Male, n (%)	50 (69)
Female, n (%)	22 (31)
Type 2 diabetes, n (%)	13 (18)
Current alcohol use, n (%)	28 (40)
Current smoking, n (%)	20 (29)
Cirrhosis, n (%)	28 (39)
BMI, kg/m^2^ (mean±SD)	27.9±7.0
Depression, n (%)^a^	5 (26)
Anxiety, n (%)^a^	8 (42)
Clinically overt fatigue, n (%)^a^	8 (42)

^a^ Detailed medical history was collected from a subgroup of subjects.

Abbreviation: CLDQ-ALD, Chronic Liver Disease Questionnaire–alcohol-related liver disease.

### Step 1: Development of CLDQ-ALD

There were 20 patients who completed the 90-item long-item selection questionnaire (26% below 45 years, 47% male, 5% with type 2 diabetes, 68% cirrhosis, 26% current alcohol use). After removal of nonimpactful items and assessment of the potential construct validity for the remaining items, 57 items were retained to yield CLDQ-ALD version 0 (Supplemental Table S1, http://links.lww.com/HC9/C356).

The CLDQ-ALD version 0 was completed by all 72 subjects included in this study (Table 1). Exploratory factor analysis of its 57 items resulted in 58% variance in those explained by 1 factor, 67% by 2 factors, 74% by 3 factors, 80% by 4 factors, 84% by 5 factors, 88% by 6 factors, 91% by 7 factors, 94% by 8 factors, 96% by 9 factors, and 99% by 10 factors. By the proportion criterion (≥95%), the resulting number of factors and, thus, domains in CLDQ-ALD was chosen to be nine.

Varimax rotation was used to calculate factor loadings for all 57 items of CLDQ-ALD. In order to meet the target instrument size, the items and their distribution into domains based on the factor loadings were subjected to an additional assessment of face and construct validity, and 17 items were further removed as non-relevant, weakly loading, or redundant.

The final CLDQ-ALD instrument has 40 items grouped into 9 domains, which were named as follows based on their primary content: Abdominal symptoms, Alcohol use disorder, Emotional well-being, Functional well-being, Fatigue, Itch, Physical well-being, Sleep disturbance, Worry (Table 2). The total CLDQ-ALD score can be calculated as an average of the 9 domain scores. Furthermore, the resulting 40-item CLDQ-ALD includes 24 items overlapping with the validated CLDQ-MASH, which are sufficient to calculate all 7 CLDQ-MASH domain scores (distinct from the CLDQ-ALD domains) (Supplemental Table S2, http://links.lww.com/HC9/C356).

**TABLE 2:** The final CLDQ-ALD structure (40 items, 9 domains)

CLDQ-ALD item description (“For how long/How often during the past 2 weeks have you been <bothered by a problem>”)	% reporting never or hardly ever bothered by the problem	Mean±SD (range 1–7)	Correlation with own domain
Domain: Abdominal symptoms			
Abdominal pain^a^	56.9	5.40±1.72	0.80
Feeling of abdominal discomfort^a^	48.6	4.88±1.86	0.80
Domain: Alcohol use disorder			
Worry about the recurrence of alcohol use	48.6	4.90±1.88	0.77
Worry about not being in active alcohol recovery	54.2	5.18±1.95	0.81
Not being aware of how to enter into alcohol recovery	62.5	5.68±1.70	0.72
Domain: Emotional well-being			
Feeling anxious^a^	31.9	4.32±1.85	0.75
Feeling unhappy^a^	36.1	4.55±1.75	0.77
Irritability^a^	38.9	4.78±1.50	0.61
Feeling depressed^a^	50.0	4.86±1.90	0.80
Problems concentrating^a^	41.7	4.66±1.97	0.74
Feeling cloudy or fuzzy in thinking	34.7	4.72±1.86	0.73
Concerns about talking to family about liver disease	44.4	4.90±1.95	0.62
Domain: Fatigue			
Tiredness or fatigue^a^	25.0	4.03±1.93	0.80
Feeling sleepy during the day^a^	36.1	4.78±1.60	0.68
Feeling the need to take naps during the day^a^	44.5	4.88±1.86	0.66
Feeling a decreased level of energy^a^	27.8	4.05±1.99	0.79
Concerned about decreased effectiveness at work	48.6	4.77±2.25	0.77
Shortness of breath^a^	59.7	5.44±1.68	0.76
Domain: Functional well-being			
Diet limitations^a^	37.5	4.65±1.90	0.64
Limitations in daily work, in and outside of the home	52.8	4.85±2.20	0.76
Worry about being unable to be employed	62.5	5.26±2.11	0.65
Concerns about engaging in social activities	38.9	4.63±2.07	0.65
Inability to work because of liver disease	51.4	4.92±2.21	0.68
Domain: Itch			
Itching (in general)^a^	58.3	5.34±1.74	0.77
Itching that disturbed sleep	73.6	5.94±1.59	0.84
Scratching that made the skin raw	80.6	6.11±1.47	0.76
Itching that impacted regular activities	79.2	6.18±1.44	0.73
Domain: Physical well-being			
Trouble walking 2 blocks, climbing 2 flights of stairs^a^	56.9	5.21±2.03	0.76
Trouble bending, lifting, or stooping^a^	48.6	4.83±2.15	0.68
Bodily pain^a^	50.0	5.22±1.77	0.74
Joint pain^a^	48.6	5.23±1.72	0.66
Domain: Sleep disturbance			
Difficulty sleeping at night^a^	26.4	3.88±1.99	0.89
Inability to fall asleep at night^a^	31.9	4.11±2.02	0.86
Difficulty obtaining a restful sleep	23.6	3.86±1.97	0.82
Domain: Worry			
Feeling that liver disease may shorten life^a^	16.7	3.58±1.83	0.76
Worry about health (in general)	22.2	3.93±1.82	0.75
Worry that symptoms will develop into major problems^a^	18.1	3.67±1.86	0.83
Worried about liver disease getting worse^a^	16.7	3.64±1.92	0.77
Worried about the impact of liver disease on family^a^	20.8	3.83±1.93	0.74
Concerns about the availability of a liver transplant	50.0	4.93±2.05	0.57

^a^ item overlapping with CLDQ-MASH (may belong to a different domain).

Abbreviation: CLDQ-ALD, Chronic Liver Disease Questionnaire–alcohol-related liver disease.

### Step 2: Validation of CLDQ-ALD

#### Internal consistency

A good to excellent internal consistency (all Cronbach’s alphas >0.85, >0.90 for 5/9 domains, none >0.95) was detected in all 9 CLDQ-ALD domains (Table 3). Additionally, after sequential one-item exclusions, the resulting Cronbach’s alphas did not change substantially (Table 3); this confirms that the items are neither too correlated with, nor too different from, other items belonging to the same domains. The greatest variability in Cronbach’s alphas over the course of one-item exclusions was found in the Alcohol use disorder domain (0.79 to 0.87 vs. pooled 0.88) (Table 3). Despite this, item-to-own-domain correlations were above +0.50 for all items, above +0.65 for 36/40 items, did not exceed +0.90 (Table 2), and all items were the highest correlated with their own CLDQ-ALD domain (discriminatory validity).

**TABLE 3:** Internal consistency of CLDQ-ALD

CLDQ-ALD domain	Cronbach alpha	Cronbach α with one item removed
Abdominal symptoms	0.89	NA
Alcohol use disorder	0.88	0.79 to 0.87
Emotional well-being	0.90	0.88 to 0.90
Fatigue	0.91	0.88 to 0.90
Functional well-being	0.86	0.81 to 0.84
Itch	0.90	0.85 to 0.89
Physical well-being	0.86	0.80 to 0.84
Sleep disturbance	0.93	0.87 to 0.93
Worry	0.90	0.87 to 0.91

Abbreviation: CLDQ-ALD, Chronic Liver Disease Questionnaire–alcohol-related liver disease.

**FIGURE 1:**
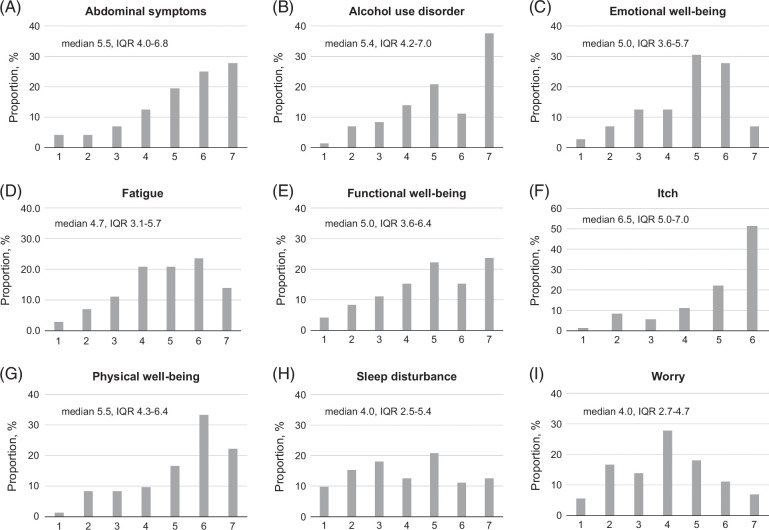
The distributions of the CLDQ-ALD domain scores. Abbreviation: CLDQ-ALD, Chronic Liver Disease Questionnaire–alcohol-associated liver disease.

The distribution of the CLDQ-ALD domain scores suggested some skewness toward higher values for most domains, with the Sleep disturbance, Worry, and Fatigue domains being relatively less skewed (Figure 1). Indeed, the lowest quartile values were at or >4.0 (center of the score range) for 4/9 domains and exceeded 3.0 for 7/9 domains, while the proportion of near-floor values (<1.5) did not exceed 10% for all domains (Figure 1). On the other hand, the median values were ≥5.0 and the highest quartile values ≥6.0 for 5/9 domains, while the proportion of the near-ceiling values (≥6.5) ranged from 7% (Emotional well-being, Worry) to 51% (Itch) (Figure 1).

#### Validity

Known-groups validity assessment of CLDQ-ALD and its discriminant function with reference to demographic and clinical parameters is summarized in Table 4. Expectedly, older patients and male patients had higher average HRQL scores in most domains, and so did subjects without type 2 diabetes, depression, or clinically overt fatigue (Table 4). The greatest HRQL impairment associated with a history of clinically overt fatigue was observed in the CLDQ-ALD Fatigue, Functional well-being, Physical well-being, and Sleep disturbance domains (mean effect size >1.0, or 16% of the score range size) (Table 4). Furthermore, subjects who reported current use of alcohol had the greatest impairment in their Alcohol use disorder domain scores (mean effect size 1.5, or 25% of the score range size) (Table 4). Finally, subjects with cirrhosis had lower scores (effect size ≥0.5, or 8%) in 5/9 domains of CLDQ-ALD: Fatigue, Functional well-being, Physical well-being, Sleep disturbance, Worry (Table 4).

**TABLE 4:** Known-groups validity of CLDQ-ALD

CLDQ-ALD domain	Age≥5 y	Age<55 years	*p*	Δ score (MCID=0.5)
Abdominal symptoms	5.86±1.23	4.73±1.81	0.0071	**1.13**
Alcohol use disorder	5.12±1.69	5.32±1.65	0.58	-0.20
Emotional well-being	4.79±1.43	4.62±1.49	0.63	0.17
Fatigue	5.18±1.40	4.37±1.59	0.0373	**0.81**
Functional well-being	5.77±1.31	4.35±1.66	0.0007	**1.42**
Itch	6.31±0.89	5.66±1.53	0.10	**0.65**
Physical well-being	5.55±1.25	4.88±1.76	0.16	**0.67**
Sleep disturbance	3.80±1.93	4.03±1.85	0.54	-0.23
Worry	4.09±1.71	3.84±1.48	0.42	0.25
Total CLDQ-ALD	5.16±0.93	4.65±1.30	0.0983	**0.51**
	Male	Female		
Abdominal symptoms	5.27±1.53	4.84±2.05	0.56	0.43
Alcohol use disorder	5.10±1.62	5.59±1.74	0.17	-0.49
Emotional well-being	4.69±1.39	4.66±1.65	0.89	0.03
Fatigue	4.88±1.31	4.16±1.97	0.17	**0.72**
Functional well-being	5.01±1.37	4.52±2.24	0.54	0.49
Itch	6.01±1.23	5.63±1.64	0.23	0.38
Physical well-being	5.44±1.32	4.40±1.98	0.0536	**1.04**
Sleep disturbance	4.12±1.81	3.57±1.97	0.25	**0.55**
Worry	3.90±1.50	3.99±1.73	0.63	-0.09
Total CLDQ-ALD	4.94±1.03	4.60±1.51	0.46	0.34
	Type 2 diabetes	No type 2 diabetes		
Abdominal symptoms	4.48±1.85	5.28±1.65	0.13	**−** **0.80**
Alcohol use disorder	4.07±1.78	5.51±1.52	0.0085	−**1.44**
Emotional well-being	3.86±1.54	4.87±1.39	0.0223	**−** **1.01**
Fatigue	4.41±1.42	4.72±1.60	0.36	−0.31
Functional well-being	4.53±1.48	4.94±1.73	0.27	−0.41
Itch	5.26±1.35	6.03±1.34	0.0277	**−0.77**
Physical well-being	4.68±1.72	5.21±1.59	0.26	**−0.53**
Sleep disturbance	3.36±2.05	4.08±1.82	0.24	**−0.72**
Worry	3.13±1.35	4.10±1.56	0.0240	**−0.97**
Total CLDQ-ALD	4.20±1.12	4.97±1.18	0.0332	**−0.77**
	Current alcohol use	No current alcohol use		
Abdominal symptoms	4.93±1.61	5.23±1.79	0.29	−0.30
Alcohol use disorder	4.33±1.47	5.82±1.53	0.0002	**−** **1.49**
Emotional well-being	4.62±1.51	4.74±1.41	0.86	−0.12
Fatigue	4.62±1.34	4.67±1.68	0.69	−0.05
Functional well-being	5.06±1.53	4.78±1.80	0.61	0.28
Itch	5.63±1.29	6.06±1.42	0.0735	−0.43
Physical well-being	5.37±1.51	4.97±1.71	0.41	0.40
Sleep disturbance	4.05±1.70	3.84±1.96	0.64	0.21
Worry	3.84±1.48	3.97±1.54	0.76	−0.13
Total CLDQ-ALD	4.72±1.13	4.90±1.24	0.46	−0.18
	Depression	No depression		
Abdominal symptoms	4.60±2.81	5.76±1.21	0.67	**−1.16**
Alcohol use disorder	5.32±2.32	6.20±0.91	0.57	**−0.88**
Emotional well-being	4.53±2.55	5.25±0.95	0.96	**−0.72**
Fatigue	4.12±2.08	5.05±1.60	0.29	**−0.93**
Functional well-being	4.84±2.43	5.41±2.02	0.78	**−0.57**
Itch	4.18±2.15	6.64±0.53	0.0031	**−** **2.46**
Physical well-being	4.42±2.65	5.56±1.69	0.28	**−** **1.14**
Sleep disturbance	4.28±2.42	4.86±1.76	0.82	**−** **0.58**
Worry	3.96±1.82	3.76±1.54	0.85	0.20
Total CLDQ-ALD	4.47±2.22	5.39±1.11	0.35	**−** **0.92**
	Clinically overfatigued	No fatigue		
Abdominal symptoms	5.13±1.88	5.69±1.71	0.55	**−** **0.56**
Alcohol use disorder	6.20±0.91	5.80±1.70	0.97	0.40
Emotional well-being	5.11±1.31	5.02±1.66	0.87	0.09
Fatigue	4.09±1.54	5.33±1.74	0.0757	**−1.24**
Functional well-being	4.33±2.47	5.93±1.53	0.14	**−1.60**
Itch	5.54±1.19	6.32±1.78	0.0171	**−0.78**
Physical well-being	4.34±1.95	5.94±1.77	0.0417	**−1.60**
Sleep disturbance	4.10±1.92	5.15±1.84	0.26	**−1.05**
Worry	3.50±1.59	4.04±1.59	0.51	**−0.54**
Total CLDQ-ALD	4.70±1.28	5.47±1.57	0.0987	**−0.77**
	Cirrhosis	No cirrhosis		
Abdominal symptoms	5.09±1.85	5.17±1.62	1.00	−0.08
Alcohol use disorder	5.54±1.75	5.07±1.59	0.19	0.47
Emotional well-being	4.64±1.44	4.72±1.49	0.75	−0.08
Fatigue	4.28±1.67	4.90±1.46	0.13	**−** **0.62**
Functional well-being	4.51±1.85	5.09±1.54	0.23	**−** **0.58**
Itch	5.98±1.51	5.84±1.28	0.37	0.14
Physical well-being	4.80±1.65	5.32±1.58	0.16	−**0.52**
Sleep disturbance	3.59±1.99	4.18±1.77	0.18	**−** **0.59**
Worry	3.55±1.44	4.17±1.60	0.17	**−** **0.62**
Total CLDQ-ALD	4.66±1.26	4.94±1.16	0.31	−0.28

Bold fonts indicate meeting MCID.

Abbreviations: CLDQ-ALD, Chronic Liver Disease Questionnaire–alcohol-associated liver disease; MCID, minimal clinically important difference.

Bold fonts indicate meeting MCID

## DISCUSSION

The CLDQ-ALD represents the first disease-specific HRQL instrument developed specifically for patients with ALD. The instrument was developed using an established methodology and includes 40 items encompassing 9 clinically meaningful domains: Abdominal symptoms, Alcohol use disorder, Emotional well-being, Functional well-being, Fatigue, Itch, Physical well-being, Sleep disturbance, and Worry.

The overall structure of CLDQ-ALD is consistent with the conceptual framework of previously validated CLDQ instruments that have been used in clinical research and clinical trials. Importantly, the CLDQ-ALD demonstrated excellent internal consistency across all domains, with Cronbach alpha values exceeding accepted thresholds while avoiding redundancy. Construct, face, and content validity were supported through factor analysis and systematic item evaluation. Furthermore, known-groups validity was clearly demonstrated, as the instrument effectively discriminated between clinically relevant subgroups, including patients with cirrhosis, clinically overt fatigue, depression, type 2 diabetes, and ongoing alcohol use.

The magnitude of differences observed across several domains, particularly Fatigue, Functional well-being, Physical well-being, Sleep disturbance, and Alcohol use disorder, was clinically meaningful and, in some instances, exceeded thresholds typically associated with minimal clinically important differences. These findings support the sensitivity of CLDQ-ALD in capturing the multidimensional burden of ALD. However, some subgroup comparisons showed trends rather than statistically significant differences; these findings warrant confirmation in larger cohorts with adequate statistical power.

An additional strength of the CLDQ-ALD is the overlap with the validated CLDQ-MASH, which is sufficient to calculate all of the CLDQ-MASH domains. This feature would enable parallel HRQL assessment in patients with mixed etiologies such as metabolic and alcohol-associated liver disease and, in general, across the spectrum of steatotic liver disease. Finally, the study incorporated input and validation from patients with both ALD cirrhosis and alcohol-associated hepatitis with active alcohol use, enhancing the applicability of the CLDQ-ALD across the spectrum of alcohol-associated liver disease, including both cirrhosis and hepatitis.

This study has several limitations. First, although the sample size was sufficient for initial psychometric development and validation, it was relatively modest and lacked broad demographic and clinical diversity, which may limit generalizability and result in some analyses being underpowered. In this context, it is important to validate the CLDQ-ALD in larger real-world cohorts, including patients across the full spectrum of alcohol-associated hepatitis severity, from mild to severe diseasse. In addition, the absence of certain clinical and sociodemographic variables precluded assessment of known-groups validity for selected patient subgroups; in particular, the formal diagnosis of alcohol use disorder was not collected as a separate variable, and neither was detailed historical alcohol consumption in the context of the established ALD diagnosis or history of “bige drinking”. Convergent validity with other validated HRQL instruments and responsiveness to longitudinal clinical change were not evaluated and should be addressed in future longitudinal studies. In addition, external validation in geographically and culturally diverse populations is required to confirm cross-cultural applicability. Finally, ceiling effects were observed in some domains (eg, Itch), which may reflect heterogeneity in symptom burden among patients with ALD; further studies in more diverse ALD populations are warranted.

In summary, we have developed and validated the CLDQ-ALD as a disease-specific instrument for assessing HRQL in patients with ALD. The instrument demonstrates strong psychometric properties and robust discriminant validity across clinically relevant subgroups. Further external and longitudinal validation in large, multinational cohorts will be important to fully establish its applicability in global clinical research and therapeutic trials involving patients with ALD.

## Supplementary Material
