# Supplementary material for: Development and validation of a disease-specific health-related quality of life questionnaire for alcohol-associated liver disease: The CLDQ-ALD
Source: Hepatol Commun. 2026 May 26;10(6):e0963. doi: 10.1097/HC9.0000000000000963 (PMC13209475; doi:10.1097/HC9.0000000000000963)
Supplement: Supplementary file 1 [file hc9-10-e0963-s001.docx]

**Supplementary Table 1**. Items included in the CLDQ-ALD version 0 after the first round of item reduction (57 items). * For the first round of item reduction, the impact scores were quantified on a scale 0-5, higher values indicate worse HRQL; ** three items were subsequently added for full concordance with CLDQ-MASH.

| Item # | Item description | mean impact score * | % “Not at all” or “A little” | % “Quite a bit” to “Very much” |
| --- | --- | --- | --- | --- |
| Q01 | Unable to eat as much as you would like. | 1.10 | **65.0%** | **15.0%** |
| Q02 | Bothered by a limitation on your diet. | 1.65 | **40.0%** | **25.0%** |
| Q06 | Limited by your liver disease in your daily work, both in and outside of the home? | 1.45 | **65.0%** | **30.0%** |
| Q07 | Having trouble walking several blocks or climbing a few flights of stairs because of your health. | 1.37 | **68.4%** | **26.3%** |
| Q08 | Having trouble bending, lifting, or stooping. | 1.47 | **63.2%** | **26.3%** |
| Q11 | Unable to be employed. | 1.37 | **68.4%** | **31.6%** |
| Q13 | Feeling anxious. | 1.63 | **52.6%** | **15.8%** |
| Q14 | Feeling unhappy. | 1.63 | **63.2%** | **21.1%** |
| Q15 | Feeling irritable. | 1.47 | **63.2%** | 10.5% |
| Q16 | Having difficulty sleeping. | 2.00 | **57.9%** | **31.6%** |
| Q17 | Having mood swings. | 1.58 | **63.2%** | **15.8%** |
| Q18 | Not enjoying life. | 1.63 | **57.9%** | **21.1%** |
| Q19 | Feeling depressed. | 1.63 | **63.2%** | **21.1%** |
| Q20 | Having problems concentrating. | 1.74 | **68.4%** | **26.3%** |
| Q21 | Unsatisfied with your life as a whole. | 1.58 | **57.9%** | **21.1%** |
| Q22 | Unable to fall asleep at night. | 1.58 | **63.2%** | **26.3%** |
| Q25 | Feeling cloudy or fuzzy in your thinking. | 1.84 | **42.1%** | **26.3%** |
| Q26 | Feeling like your liver disease may shorten your life. | 3.21 | **10.5%** | **57.9%** |
| Q30 | Feeling an emotional strain or stress in your relationship as a result of your liver disease. | 1.58 | **52.6%** | **26.3%** |
| Q34 | Feeling frustrated by having liver disease. | 2.79 | **21.1%** | **47.4%** |
| Q36 | Worried about your general health. | 2.21 | **42.1%** | **42.1%** |
| Q37 | Worried about recurrence of alcohol use. | 1.37 | **68.4%** | **21.1%** |
| Q40 | Worried that your symptoms will develop into major problems. | 2.84 | **15.8%** | **57.9%** |
| Q41 | Worried about your alcohol related liver disease getting worse. | 2.84 | **21.1%** | **57.9%** |
| Q42 | Worried about the impact your liver disease has on your family. | 2.79 | **21.1%** | **63.2%** |
| Q43 | Worried about never feeling better. | 1.79 | **47.4%** | **26.3%** |
| Q45 | Concerned about the availability of a liver if you need a liver transplant. | 2.05 | **47.4%** | **36.8%** |
| Q46 | Concerned about telling your family you have alcohol-related liver disease. | 1.37 | **52.6%** | **21.1%** |
| Q47 | Unable to perform usual social activities with your friends and family because of your liver disease. | 1.47 | **63.2%** | **26.3%** |
| Q61 | Troubled by a feeling of abdominal bloating. | 2.05 | **42.1%** | **26.3%** |
| Q62 | Troubled by abdominal pain. | 1.05 | 73.7% | 10.5% |
| Q63 | Troubled by abdominal discomfort. | 1.53 | **68.4%** | **21.1%** |
| Q64 | Being tired or fatigued. | 2.26 | **31.6%** | **31.6%** |
| Q65 | Feeling sleepy during the day. | 2.05 | **42.1%** | **36.8%** |
| Q66 | Feeling drowsy. | 1.74 | **57.9%** | **26.3%** |
| Q67 | Not having enough energy to do the things you want to do. | 2.05 | **36.8%** | **26.3%** |
| Q68 | Taking naps (5 min. or longer) during the day. | 1.32 | **63.2%** | **26.3%** |
| Q71 | Difficulty obtaining a restful sleep. | 2.16 | **42.1%** | **47.4%** |
| Q72 | Decreased strength. | 2.00 | **52.6%** | **36.8%** |
| Q73 | Decreased level of energy. | 2.42 | **36.8%** | **47.4%** |
| Q74 | Experiencing bodily pain. | 1.42 | **57.9%** | **26.3%** |
| Q75 | Having muscle cramps. | 1.53 | **52.6%** | **21.1%** |
| Q76 | Experiencing joint pain. | 1.53 | **52.6%** | **26.3%** |
| Q77 | Frequently troubled by itching. | 1.42 | **68.4%** | **21.1%** |
| Q78 | Itching that disturbs your sleep. | 0.84 | 84.2% | 10.5% |
| Q79 | Scratching that makes your skin raw. | 0.63 | 89.5% | 5.3% |
| Q81 | Itching that impacts your regular activities. | 0.47 | 89.5% | 5.3% |
| Q82 | Concerned about the cost of medications for your liver disease. | 1.37 | **68.4%** | **15.8%** |
| Q83 | Having financial problems because of medical expenses due to your liver disease. | 1.58 | **68.4%** | **26.3%** |
| Q84 | Being unable to work because of your liver disease. | 1.47 | **68.4%** | **26.3%** |
| Q85 | Concerned that having liver disease will decrease your effectiveness at work. | 1.68 | **63.2%** | **36.8%** |
| Q86 | Worried about not being in active alcohol recovery. | 0.89 | 73.7% | 10.5% |
| Q87 | Worried because you do not know how to enter into alcohol recovery. | 0.32 | 94.7% | 5.3% |
| Q90 | Reluctant/concerned about engaging in social activities because of the fear of recurrence of alcohol use. | 1.21 | **68.4%** | **15.8%** |
| na | Shortness of breath been a problem in your daily activities | Na | Na | Na |
| na | Trouble lifting or carrying heavy objects | Na | Na | Na |
| na | Dry mouth | Na | Na | Na |

**Supplementary Table 2**. Overlap between CLDQ-ALD and CLDQ-MASH (sorted by CLDQ-MASH domain).

| Item description (“For how long / How often during the past two weeks have you been <bothered by a problem>”) | CLDQ-ALD domain | CLDQ-MASH domain |
| --- | --- | --- |
| Shortness of breath | Fatigue | Activity |
| Trouble walking two blocks, climbing two flights of stairs | Physical well-being | Activity |
| Trouble bending, lifting, or stooping | Physical well-being | Activity |
| Decreased strength | Na | Activity |
| Trouble lifting or carrying heavy objects | Na | Activity |
| Abdominal pain | Abdominal symptoms | Digestive symptoms |
| Feeling of abdominal discomfort | Abdominal symptoms | Digestive symptoms |
| Diet limitations | Functional well-being | Digestive symptoms |
| Feeling of abdominal bloating | Na | Digestive symptoms |
| Not being able to eat as much as one would like | Na | Digestive symptoms |
| Feeling anxious | Emotional well-being | Emotional well-being |
| Feeling unhappy | Emotional well-being | Emotional well-being |
| Irritability | Emotional well-being | Emotional well-being |
| Feeling depressed | Emotional well-being | Emotional well-being |
| Problems concentrating | Emotional well-being | Emotional well-being |
| Mood swings | Na | Emotional well-being |
| Not enjoying life | Na | Emotional well-being |
| Tiredness or fatigue | Fatigue | Fatigue |
| Feeling sleepy during the day | Fatigue | Fatigue |
| Feeling the need to take naps during the day | Fatigue | Fatigue |
| Feeling a decreased level of energy | Fatigue | Fatigue |
| Feeling drowsy | Na | Fatigue |
| Difficulty sleeping at night | Sleep disturbance | Sleep disturbance |
| Inability to fall asleep at night | Sleep disturbance | Sleep disturbance |
| Itching (in general) | Itch | Systemic symptoms |
| Bodily pain | Physical well-being | Systemic symptoms |
| Joint pain | Physical well-being | Systemic symptoms |
| Muscle cramps | Na | Systemic symptoms |
| Dry mouth | Na | Systemic symptoms |
| Feeling that liver disease may shorten life | Worry | Worry |
| Worry that symptoms will develop into major problems | Worry | Worry |
| Worried about liver disease getting worse | Worry | Worry |
| Worried about the impact of liver disease on family | Worry | Worry |
| Worried about never feeling any better | Na | Worry |
| Feeling distressed by having liver disease | Na | Worry |
| Worry about recurrence of alcohol use | Alcohol use disorder | Na |
| Worry about not being in active alcohol recovery | Alcohol use disorder | Na |
| Not being aware how to enter into alcohol recovery | Alcohol use disorder | Na |
| Feeling cloudy or fuzzy in thinking | Emotional well-being | Na |
| Concerns about talking to family about liver disease | Emotional well-being | Na |
| Concerned about decreased effectiveness at work | Fatigue | Na |
| Limitations in daily work, in and outside of the home | Functional well-being | Na |
| Worry about being unable to be employed | Functional well-being | Na |
| Concerns about engaging in social activities | Functional well-being | Na |
| Inability to work because of liver disease | Functional well-being | Na |
| Itching that disturbed sleep | Itch | Na |
| Scratching that made skin raw | Itch | Na |
| Itching that impacted regular activities | Itch | Na |
| Difficulty obtaining a restful sleep | Sleep disturbance | Na |
| Worry about health (in general) | Worry | Na |
| Concerns about the availability of a liver transplant | Worry | Na |
